# Supplementary material for: Comparative multiomics analysis of cell physiological state after culture in a basket bioreactor
Source: Sci Rep. 2022 Nov 23;12:20161. doi: 10.1038/s41598-022-24687-4 (PMC9686226; doi:10.1038/s41598-022-24687-4)
Supplement: Supplementary file 1 — Supplementary Information 1. [file 41598_2022_24687_MOESM1_ESM.zip › raw data/Transcriptomic raw data/6.Enrichment/1.GO/TE1vsTC1/ALL/TE1vsTC1.all.GObp_DAG.pdf]

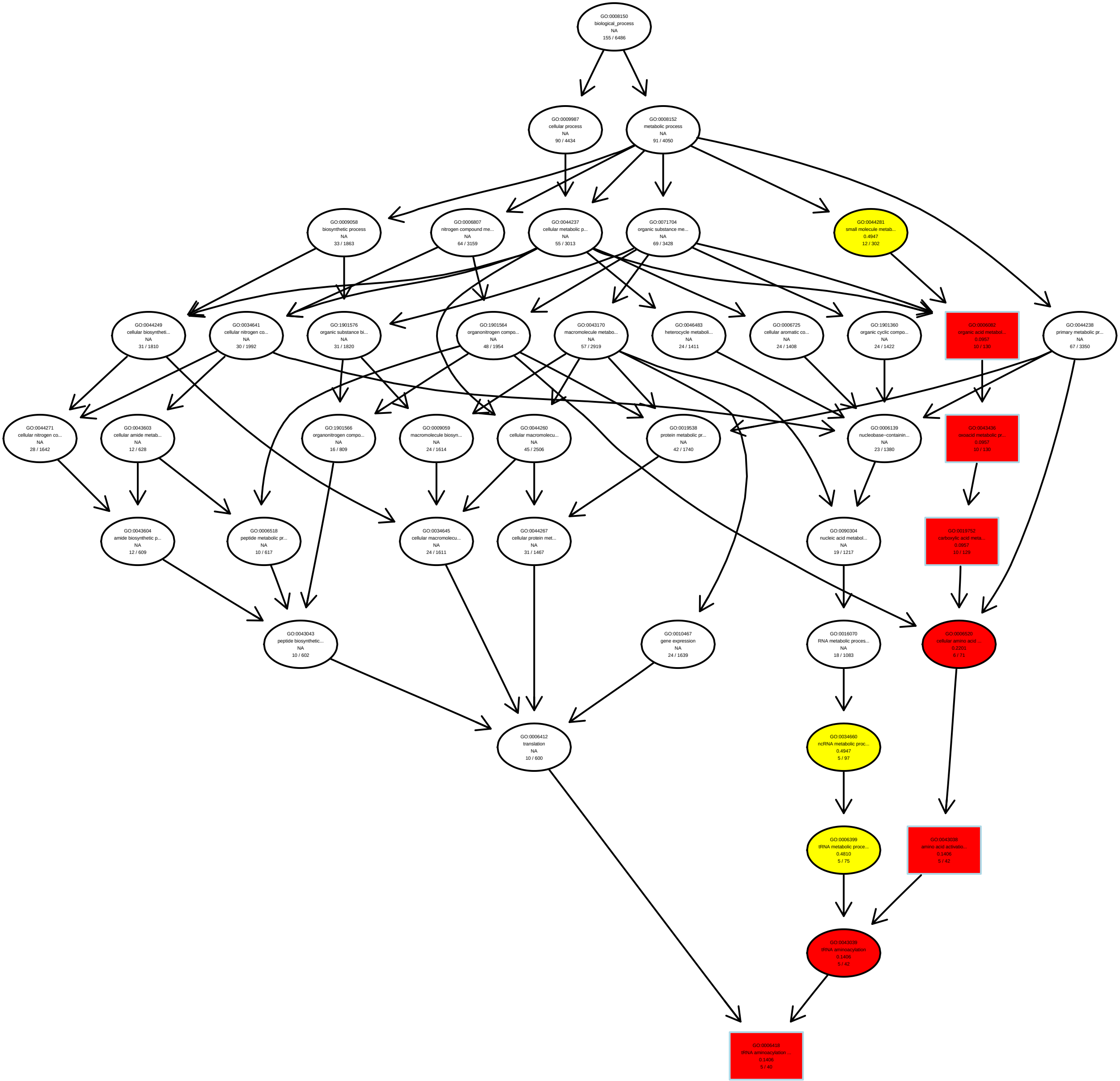
GO:0008150
biological_process
NA
155 / 6486
GO:0009987
cellular process
NA
90 / 4434
GO:0008152
metabolic process
NA
91 / 4050
GO:0009058
biosynthetic process
NA
33 / 1863
GO:0006807
nitrogen compound me...
NA
64 / 3159
GO:0044237
cellular metabolic p...
NA
55 / 3013
GO:0071704
organic substance me...
NA
69 / 3428
GO:0044281
small molecule metab...
0.4947
12 / 302
GO:0044249
cellular biosyntheti...
NA
31 / 1810
GO:0034641
cellular nitrogen co...
NA
30 / 1992
GO:1901576
organic substance bi...
NA
31 / 1820
GO:1901564
organonitrogen compo...
NA
48 / 1954
GO:0043170
macromolecule metabo...
NA
57 / 2919
GO:0046483
heterocycle metaboli...
NA
24 / 1411
GO:0006725
cellular aromatic co...
NA
24 / 1408
GO:1901360
organic cyclic compo...
NA
24 / 1422
GO:0006082
organic acid metabol...
0.0957
10 / 130
GO:0044238
primary metabolic pr...
NA
67 / 3350
GO:0044271
cellular nitrogen co...
NA
28 / 1642
GO:0043603
cellular amide metab...
NA
12 / 628
GO:1901566
organonitrogen compo...
NA
16 / 809
GO:0009059
macromolecule biosyn...
NA
24 / 1614
GO:0044260
cellular macromolecu...
NA
45 / 2506
GO:0019538
protein metabolic pr...
NA
42 / 1740
GO:0006139
nucleobase−containin...
NA
23 / 1380
GO:0043436
oxoacid metabolic pr...
0.0957
10 / 130
GO:0043604
amide biosynthetic p...
NA
12 / 609
GO:0006518
peptide metabolic pr...
NA
10 / 617
GO:0034645
cellular macromolecu...
NA
24 / 1611
GO:0044267
cellular protein met...
NA
31 / 1467
GO:0090304
nucleic acid metabol...
NA
19 / 1217
GO:0019752
carboxylic acid meta...
0.0957
10 / 129
GO:0043043
peptide biosynthetic...
NA
10 / 602
GO:0010467
gene expression
NA
24 / 1639
GO:0016070
RNA metabolic proces...
NA
18 / 1083
GO:0006520
cellular amino acid ...
0.2201
6 / 71
GO:0006412
translation
NA
10 / 600
GO:0034660
ncRNA metabolic proc...
0.4947
5 / 97
GO:0006399
tRNA metabolic proce...
0.4810
5 / 75
GO:0043038
amino acid activatio...
0.1406
5 / 42
GO:0043039
tRNA aminoacylation
0.1406
5 / 42
GO:0006418
tRNA aminoacylation ...
0.1406
5 / 40
